# The Hospital. Nursing Section

**Published:** 1904-10-22

**Authors:** 


### The Hospital.

### Nursing Section.

Contributions for this Section of "THE HOSPITAL" should be addressed to the EDITOR, "THE HOSPITAL," NURSING SECTION, 28 & 29 Southampton Street, Strand, London, W.C.

No. 943.—VOL. XXXVII. *SATURDAY, OCTOBER* 22, 1904.

### Notes on News from the Nursing World.

### EASILY-EARNED HONOURS.

THE Emperor of Japan has conferred the order of the Crown on the American nurses serving in the Japanese hospitals, and the American nurses have quitted Japan. We are supplied this week by a member of the Order of Spanish-American War Nurses with an account—from which we take extracts—written by Dr. Anita Newcomb McGee, the president of the Order, who went to Japan in charge of the volunteers, and Miss King, a Philadephian nurse, of their experiences in the Far East, which will be read with interest. They entirely bear out the statement published in the *New York Medical Record*, to which we referred a fortnight ago, that while the assistance of foreign nurses was not desired in Japan, the contingent of volunteers from the United States, who persisted in going, were received and treated with the utmost courtesy. From the details which we are now able to give, as far as space will afford, it appears that one of the foreigners managed to obtain a fair knowledge of the Japanese language, and that the others picked up a few stray words, and that all of them conformed "as much as possible" to the Japanese way of doing things. Having arrived in Japan, it was naturally considered advisable by the military authorities that an effort should be made to employ them, and we do not doubt that being fully trained they performed the duties assigned to them in the Army Reserve Hospital at Hiroshima with diligence and devotion. The remarkable point is that at the very time, as the Philadelphia nurse says, when the heaviest of the work was coming, their services were dispensed with. They had left Yokohama for the States long before the battles involving the greatest bloodshed were fought. With their blushing honours thick upon them, they were in a position to tell their friends in America how successfully they had taught "a lesson of independence to Japanese women," while the civilised world was ringing with the story of the carnage which was going on in the country they had left. We hope that in turn they have been taught a lesson of modesty, and that the Crowns which have been politely bestowed upon them by the Emperor of Japan will reconcile them to the mortification of being sent back to the States very much in the character of returned empties.

### QUEEN ALEXANDRA'S MILITARY NURSING SERVICE.

WE are officially informed that Miss N. Blew has been appointed staff nurse in Queen Alexandra's Imperial Military Nursing Service, and posted to Portsmouth; that Miss A. C. Jacob and Miss A. R. Rose-Innes have resigned their appointments as sisters on their marriage; that Miss A. Garriock, Royal Red Cross matron, has been transferred from Alton to s.s. *Plassy* for Indian transport duty; and that Miss A. FitzGerald, sister, has been transferred from Aldershot to s.s. *Plassy* for Indian transport duty.

### PRINCESS CHRISTIAN AT PRETORIA.

AN interesting and pleasant little function took place at the Military Hospital, Pretoria, on Sunday afternoon, September 25, when Princess Christian and her daughter visited the Hospital train, the wards, and the sisters' quarters. The Royal Party arrived at the Hospital at 3.45 P.M., and were met at the "Princess Christian Hospital Train" by the officials, including the matron. After an inspection of the train, which lies at the entrance to the Camp on the Delagoa Bay line, a tour of the wards was made. The Princess spoke kindly to many of the patients, and manifested special interest in the little ones in the Women's and Children's Ward. The Royal Party then proceeded to the sisters' quarters where the nursing staff lined up on either side of the entrance to the mess tent in order to welcome their president. During tea her Royal Highness conversed with many of the sisters, and the pleasant visit terminated at the close of an hour.

### THE LADY SUPERIOR OF THE PASTEUR HOSPITAL ON ENGLISH NURSING.

IT is noteworthy that the Lady Superior of the Pasteur Hospital in Paris, with whom one of our contributors had a chat while she was in London, states that she was particularly struck with the extreme comfort, "almost luxury" of the London hospitals which she visited. She was also impressed with the care and attention which seemed to be given to both patients and nurses. Another interesting point is that she expressed a hope to be able in future to train some of the young sisters who belong to the order of which she is a member "more as English probationers are trained." This shows a commendable desire to take advantage of all that can be learnt, and we certainly share the opinion of the Lady Superior that sisters of charity "can be taught to nurse quite as well as other women." It is usually only when they have not been taught that they are found deficient.

### GUY'S HOSPITAL TRAINED NURSES' INSTITUTION.

THE nineteenth annual report of Guy's Hospital Institution for Trained Nurses, which has just been issued, shows that a larger income was earned and a larger bonus distributed than in any previous year. This, as the report says, is the more satisfactory because 1903 was a year of unusual healthiness, and it justifies the conclusion that the high quality of the nurses which the institution provides is increasingly appreciated by the public. The work actually done also compares very favourably with the previous year, 1,019 patients having been nursed, 1,145 applications received, as against 903 patients and 1,256 applications in 1902. Forty eight of the nurses shared in the bonus of nearly £2,000, which was apportioned at the rate of £6 a share, and invested on the nurses' behalf in the Royal National Pension Fund. The excess of assets over liabilities at the close of the year was £2,169 in comparison with £2,105 in 1902.

### THE ADEQUATE SUPPLY OF MIDWIVES.

An important meeting under the auspices of the Association for promoting the Training and Supply of Midwives will be held at the Westminster Palace Hotel on Friday, November 4th, at 2.45 p.m. The object in view is to place before the public the urgent need of training an adequate supply of midwives for the poor in order to meet the requirements of the Act of 1902. Her Royal Highness, Princess Henry of Battenberg, has graciously consented to be present on the occasion, the chair will be taken by the Archbishop of Canterbury, and the speakers, in addition to the Primate, will include Lord Brassey, the Chief Rabbi, Dr. F. H. Champneys, and Mrs. Humphry Ward.

### THE POOR-LAW INFIRMARY NURSE.

At the close of his "Reminiscences of Workhouse Nursing," which Major Ballantine contributes to our columns to day, the late master of Crumpsall contends that, instead of the well-trained Poor-law infirmary nurse being placed at a disadvantage as compared with the nurse trained at a general hospital, she enjoys the advantage. We recently observed that a hospital-trained nurse often lacks the common sense and kindliness which Poor-law infirmary nurses gain as a part of their training; and Major Ballantine maintains that Poor-law infirmary nurses no longer labour under the drawback of want of experience in surgical cases, while they are called upon to attend patients suffering from a greater variety of ailments. But it must be remembered that the experience of a considerable number of them has not been obtained under the conditions indicated by him. There is all the difference in the world between the training at the large up-to-date workhouse infirmaries and that at some of the smaller institutions which nevertheless arm a nurse with a certificate that she is qualified to pursue her calling.

### DISMISSAL FOR DISOBEDIENCE.

At the Brighton County Court on Friday last a nurse sued the superintendent of the London and Brighton Association of Nurses for £46 8s. 10d., as damages for alleged wrongful dismissal. The plaintiff stated that she entered the employ of the defendant in September 1903. The defendant sent her a copy of the rules of the association. Up to June last she was on several occasions absent for more than an hour at a time without the defendant's consent. The nurse who was next for duty had to report herself every hour, as plaintiff always did when it was her turn. In June she went home for a few days, and arranged that she should be the last for duty, with eight other nurses before her. On June 19th she went out at 2 p.m. and returned at 8 p.m. On her return she heard that defendant had lost a case through her absence, and that she was very angry about it. The plaintiff then tendered her resignation, which defendant refused to accept, and told her to leave at once, because she had broken the rules. Defendant paid her a quarter's salary, £8 15s. 8d. The plaintiff added that she had been unable to get any work since owing to her having been summarily dismissed without a testimonial. Several nurses stated that the rule as to not being out for more than an hour was only observed by the nurse next for duty. The defendant denied that she had ever abrogated the rule as to the nurses being absent for more than an hour together without permission. After the plaintiff came back from her visit to her home she told her that she must take her place as next for duty. On the next day, however, she went out at two o'clock and did not return until eight. She was wanted whilst she was out, and when she returned defendant told her that she ought not to have been away more than an hour without permission. Every nurse, she said, had to report herself every hour, unless she had special permission. After hearing the evidence of several nurses for the defence, the judge told the jury that plaintiff would not be entitled to damages for injury to her character or for not having a testimonial, and was only entitled to a month's wages in lieu of notice. The jury found accordingly, with costs.

### ROYAL NATIONAL PENSION FUND FOR NURSES.

The Secretary of the Junius S. Morgan Benevolent Fund asks our permission to remind those members of the Pension Fund who have forgotten to send their contribution of 1s. to their Benevolent Fund that the accounts for the year are about to be made up. She goes on to say: "Every year we are asked to help a larger number of cases of temporary or permanent distress, and we find it a hard matter to do all that is needed. If every Pension Fund nurse would give only 1s. yearly to help sister nurses in time of trouble, our difficulty would cease." Contributions should be sent to the Secretary of the Pension Fund, 28 Finsbury Pavement.

### PUBLICANS AND DISTRICT NURSING.

The annual meeting of the Appleby and Dowgate District Nursing Society, affiliated to Queen Victoria Jubilee Institute, was held the other day, Lady Hothfield presiding. The nurse, whose services were spoken of in terms of warm appreciation, attended 94 cases, paying 2,653 visits. The treasurer read the balance-sheet, which was quite satisfactory, there being, besides the amount already invested, a balance in hand of £25 5s. There were 24 new subscribers since last year, including a joint donation of £5 5s. from the publicans of the town, who expressed their willingness, should any deficiency arise, to increase the amount.

### THE PALMISTRY CASE.

The person who was found guilty along with her husband at Clerkenwell Sessions on Thursday, October 6th, for obtaining money under false pretences by pretending to tell fortunes, whilst giving her evidence, stated that she was formerly a "sister" or "trained nurse" at St. Bartholomew's Hospital. We understand that within the last 20 years no such person has ever been a sister at St. Bartholomew's, neither does her name appear in the directory of past and present St. Bartholomew's trained nurses.

### EXTENSION OF THE BRISTOL INFIRMARY NURSES' HOME.

A satisfactory announcement was made by Sir George White at the quarterly meeting of the Board of the Bristol Royal Infirmary. Although there is a very fine nurses' home attached to the infirmary, the accommodation, which is for 99 nurses, has for some time been insufficient, and has constituted a serious difficulty. It has now, however, the President intimated, been solved by the acquisition of a large house and garden of about a quarter of an acre in Bedford Place, nominally adjoining the home. This will mean not only ample provision for a large nursing staff, but also a beautiful addition to the nurses' present garden. It has, we are glad to notice, been arranged to purchase some property which can be turned to account in considerably reducing the distance between the infirmary and the home.

### THE LOCAL GOVERNMENT BOARD AND THE MATRON OF LEWISHAM INFIRMARY.

The Lewisham Guardians, who objected to the appointment of Miss Henrietta Strick as matron of Lewisham Infirmary on the ground that her training was not in strict accordance with the standard, elicited a reply which, from their point of view, justifies their action. The Local Government Board insist that a three years' training certificate from the Lewisham guardians is requisite. They do not, however, explain why, when the guardians appointed her a sister they ratified the appointment, though the qualifications are identical with those required for matron. The inference is that no one protested against it. However, the objectors to Miss Strick's promotion have now consented to a settlement of the issue which, we suppose, will be approved by the Local Government Board. The medical superintendent is to give her a certificate for her first year's training at Lewisham, the first year's training to be added to the two years at Leicester; or Miss Strick is to be allowed to present herself to the present examiner, and on a satisfactory report being received from him, a three years' certificate will be granted to her in the form adopted by the guardians in 1898. We think that the importance of the office of matron justifies the most searching inquiry as to qualifications, but in this case there has been unnecessary fuss about a technical point.

### ONE NIGHT NURSE FOR THREE HOSPITALS.

The condition of workhouse nursing at Kidderminster leaves much to be desired. At a recent inquest on the body of an old woman who died in the workhouse infirmary from the results of injuries sustained in a fall in one of the wards, it transpired in evidence that there was but one night nurse in charge of three hospitals containing about 150 patients. Another extraordinary feature is that the medical officer of the workhouse, who states that the nurse was supposed to visit all three hospitals and go through all the wards several times in the night, said he was inclined to hold that one nurse was sufficient, because there were wards-women, who were partly able-bodied, in each ward. The coroner very properly observed that one night nurse in each hospital was required, and condemned the employment of inmates as assistant nurses on the ground that if they were able-bodied and able-minded persons they would scarcely be in the workhouse at all. As a further instance of mismanagement the wards-woman who "helped" the deceased patient into bed again, did not use the bell, which was the ordinary method of calling the night nurse, because she thought that it was out of order.

### NURSES' MISSIONARY UNION.

There was an interesting gathering in connection with the Nurses' Missionary Union on Friday evening last. Two of its members are sailing shortly to take up missionary work in distant lands. Miss Reed, who was sister at the East London Hospital, besides having held other posts in the nursing world, is going out under the auspices of the Church Missionary Society to the hospital in Toro, Uganda; and Miss Cottis, who was trained at the Seamen's Hospital, Greenwich, is to serve at the hospital in Nasik, India, built by a rich Brahmin lady and recently handed over to the Zenana and Bible Medical Mission. Lady Wingate, in the absence of the Hon. Lady Tracey, who lent her house in Sloane Gardens, received the nurses and friends, who assembled in goodly number to bid farewell to the two ladies. Sir Andrew Wingate, K.C.I E., took the chair and gave an interesting account of Nasik and of the progress of missionary work in that bigoted, sacred Hindu city. He also alluded to the remarkable work of Miss Harvey, who has been labouring in the city over twenty years without once coming home on furlough. Both of the outgoing nurses spoke a few words to their fellow-nurses, and Miss Reed said that she hoped this would not be a "good-bye" for very long, but that soon she would meet in Africa some who would come out to help in the great work. Miss Fairfield explained the objects of the Nurses' Missionary Union and stated that it was proving its usefulness by bringing hospital nurses into touch with the various missionary societies; and Colonel Oldham gave the final address of farewell.

### A FORWARD MOVE IN DEVON.

On Friday next week a meeting will be held at the Castle, Exeter, convened by Lady Ebrington, for the purpose of taking steps to overcome some of the difficulties which had manifested themselves since the enactment of the Midwives Act of 1902. It is proposed to form an association to be affiliated with the Queen Victoria Jubilee Institution, for the purpose of encouraging and developing all classes of district nurses in Devon, and to meet the requirements of the Midwives Act, as far as possible, by the provision of certificated midwives. The Lord-Lieutenant, Lord Ebrington, will preside at the gathering, and Miss Amy Hughes will be one of the speakers. The scheme, under such auspices, and with the support of the principal county families, will have an excellent inauguration, and the £500 a year which is needed for the purpose of training and other details incidental to the improvement of nursing, should not be difficult to secure.

### SHORT ITEMS.

On Thursday last week there passed away a familiar figure in the nursing world in the person of Miss Sarah Blomfield. She was for eleven years—up to July 1901—head of the East End Mothers' Home, where she did much excellent work both amongst the poor and in teaching the special branch of work for which that hospital exists. She knew her work thoroughly and was a splendid organiser.

### The Nursing Outlook.

"From magnanimity, all fear above;
From nobler recompense, above applause,
Which owes to man's short outlook all its charm."

### THE JUBILEE OF FLORENCE NIGHTINGALE.

On October 21st, 1854, Miss Florence Nightingale left England with a staff of 37 nurses for the Crimea. Friday, October 21st, 1904, may therefore be regarded as the day on which Florence Nightingale will attain her jubilee.

Few people nowadays realise the condition of affairs 50 years ago. In 1847 Sir Edward Parry published a request for nurses for Haslar Naval Hospital. They were to be trained at the German Hospital, Dalston, for six months, and, if possible, at the Institute for Deaconesses at Kaiserswerth. There was not one volunteer in answer to this appeal; not a single woman in England who at that time would volunteer to train as a nurse. What a striking instance of the lack of enthusiasm for the work of nursing before Florence Nightingale became a heroine in the cause.

In 1851 Miss Nightingale entered the institute at Kaiserswerth. After a year's training Miss Nightingale acted as superintendent of the Home for Gentlewomen in Harley Street, and in the same year she published her first "Notes on Hospitals."

On March 28th, 1854, war was declared against Russia by England and France. It soon became apparent that there were no proper arrangements for nursing the sick and wounded soldiers in the Crimea. Miss Nightingale was called upon to organise such a service, and she arrived with her staff at Scutari on November 5th, 1854. This is not the place to enter into details showing the invaluable services she rendered to the Army and the nation in this connection. Important as the service undoubtedly was, its importance is as nothing compared with the immensity of the value to the human race of the consequences which followed from that work, by improving the nursing of the sick everywhere throughout the world. Miss Nightingale's special services to the nation in the Crimea were commemorated by the establishment of the Nightingale Fund at a meeting at Willis' Rooms in November 1855. The subscriptions closed on April 24th, 1857, and amounted to upwards of £44,000. The wisdom displayed by Miss Nightingale in her scheme for the training of nurses is proved by the fact that the nurses of the Nightingale Fund are everywhere, and deservedly, welcome to-day as probably the most efficient and acceptable attendants on the sick which the world possesses.

In June 1860 the Nightingale School for the training of nurses was opened in connection with St. Thomas's Hospital through the generosity of Miss Nightingale, who devoted the whole of the public subscriptions to this work. In 1862 Miss Nightingale established a training school for midwives in connection with King's College Hospital, and from that time to the present Miss Nightingale has continuously devoted her energies to the effort to improve nursing throughout the world.

The condition of affairs in 1862 is well shown by the following extract from a letter of Miss Nightingale written to the Mayor of Liverpool to further the establishment of a training school and home for nurses in connection with the Royal Infirmary of that city:—

"Sickness is everywhere; death is everywhere. But hardly anywhere is the training necessary to teach women how to relieve sickness, to delay death. We consider a long education and discipline absolutely necessary to train our medical men; we consider hardly any training at all necessary for our nurses, although how often does our medical man himself tell us, 'I can do nothing for you unless your nurse will carry out what I say.'" Here was the actual truth, tersely stated in simple language, which appealed to everybody—so it has ever been with Miss Nightingale and her utterances. It has been our privilege to spend many years, with the assistance of the Government Departments of this country and other nations, in collecting all the facts relating to the care, treatment, and nursing of the sick in every portion of the world. In the course of our researches we have everywhere found striking evidence of the irresistible forces created by the example of Florence Nightingale, of its influence upon peoples and Governments. It is but the bare truth to declare that there is not a single British Colony, nor a single nation with any pretensions to civilisation, where Florence Nightingale's influence has not worked for good by improving the conditions of the sick and the quality of the nursing. We believe it to be a fact that Florence Nightingale owed a great deal to the help and counsel of her late sister, Lady Harry Verney, who had one of the brightest minds and most attractive characters England possessed. It was indeed an inspiration and a glorious privilege to spend an hour with Lady Verney, and to realise that the mind and soul are absolutely independent of bodily suffering, and may triumph over all the most terrible infirmities to which the human frame is liable. In work such as that undertaken by Florence Nightingale, the sympathy, support, and counsel of such a sister must have proved a tower of strength.

But to Florence Nightingale must ever belong the honour for the results of her work which can never be forgotten so long as the world endures. She has had the privilege of living to see the effects of her teaching and example upon the peoples of the earth. She must know that under Providence the sick owe her more, probably, than they owe even to the great profession of medicine. There are many cases where life may depend upon the quality of the nursing, and the modifications and advancement in treatment due to the recognition of this fact must have proved potent in the saving of life.

From the highest to the humblest all owe gratitude to Florence Nightingale, and there cannot be a single human soul who will not render praise and good wishes on her jubilee to the heroine of the nursing world.

### Lectures Upon the Nursing of Mental Diseases.

By Robert Jones, M.D.Lond., B.S., F.R.C.S.Eng., M.R.C.P.Lond., Resident Physician and Superintendent of the London County Asylum, Claybury.

LECTURE XX. (*continued from page* 19).

As to the treatment of the respiratory affections enumerated, the nurse should never allow a catarrh or a cold to be neglected. The patient should be kept indoors and in an equable temperature, with plenty of good and nourishing food. A few days in bed may save complications, and extension of the catarrh down the bronchi. For laryngitis the bronchitis kettle may afford much relief, the air being kept moist and the temperature of the room at 65°. Inhalation, if the patient's mental condition permits, will also give relief. If the case be serious, surgical relief by means of tracheotomy may be necessary, and the nurse will have to prepare the dressings and the instruments and be capable of cleaning the tube. For bronchitis the same surroundings are necessary, draughts and chills are to be avoided, and the temperature of the room must be equable. The patient's shoulders are to be raised up in bed, if preferred, and paper expectoration pots should be used so that they can be destroyed. The various remedies are inhalations, which are prepared according to instructions, poultices, and cough medicines. The effect of medicines must be observed and recorded by the nurse, as stupor and lividity may appear and mental symptoms such as delirium aggravated. Fluid nourishment and stimulants are to be given as ordered. For asthma a cup of coffee may relieve an attack, or the burning of nitre paper, but a good aperient may prove more effective than any other remedy.

Pneumonia is one of the diseases that especially needs good nursing. The patient is kept in bed, the room must be quiet and the bedclothes light but warm. Pain is relieved by ice or mustard poultices in the early stages, or by large hot linseed meal poultices, or a cotton-wool jacket after the first stage. A receptacle for the sputum should be kept and a bed pan used so that the patient does not get out of bed, and great care must be exercised to prevent bedsores. Ice, or iced drinks, lemonade, or gruel should be given to quench the thirst and fluid food must be regularly and freely administered. It is for this disease, especially for the nervous symptoms, that stimulants are so useful. The nurse must report any change in the appearance of the sputum, the pulse, temperature and respirations. Pleurisy may require the use of an aspirator to tap the chest wall with, and the nurse must be careful about frequent changes in the dressings if pus is withdrawn, as in such a case the chest is probably opened under an anæsthetic, and a tube kept in the opening for the pus to drain through. These cases of empyema usually require much attention to prevent blood-poisoning. For pulmonary consumption the nurse must exercise constant care that all spoons, cups, forks, etc., used by the patient are properly disinfected, and of all disinfectants the most efficient and least harmful is boiling water. The utensils must be boiled after use, and all sputum, if cough occurs and expectoration takes place, must be burnt. This is often difficult with the insane as many of them spit about the room, bedding, and bedclothes. The room is kept clean by wet scrubbing, and all dusting done with a wet cloth to avoid raising the dust and with it millions of infective bacilli. The patients should be in the open air as much as possible, and when in a room it should be well ventilated, there being plenty of fresh air without causing a draught. The dietary should include milk, cream, and butter, or, failing these fats, cod-liver oil will be found very suitable, and it can often be well digested. If diarrhœa be present the stools must be disinfected, also all soiled linen or bedding. It is often possible to relieve pain in the chest by hot poultices or fomentations and a simple linctus will relieve the cough when present. The flannels must be changed if there is profuse night sweating, and barley-water, egg-albumen water, or lemonade must be given to allay thirst. Tepid sponging relieves the high temperature, and a little fountain or spray of eau de Cologne or other scent will often give patients much gratification. For the spitting of blood, also called hæmoptysis, ice to suck, cold drinks, and absolute quietness in the recumbent position are the best remedies within the power of the nurse. Even the insane are greatly frightened at the sight of their blood and there is often considerable shock, and whilst the doctor may meet this by an injection of morphia, the tact of the nurse will do much to preserve the quiet and restful demeanour so necessary for such cases.

As to diseases of the circulatory system, there are three cardinal symptoms of heart affections—viz., dropsy, difficulty of breathing, and lividity; the latter is present mostly in cases of mitral disease and in affections of the right side of the heart; it is only a sign of general congestion and engorgement caused by heart weakness. It is absent in the most dangerous form of heart disease, viz, aortic valve disease, a form which causes sudden death; the patient in this form being often white and blanched and liable to sudden faintness which may be fatal. Palpitation is a *symptom* of functional as well as of organic heart disease, but it is not a *sign* as it may arise from indigestion, anxiety, or worry, over smoking and other causes. The dropsy of heart disease commences usually in the most distant parts, and it is termed *œdema*. If dropsy begins in the face and the eyelids become puffy, kidney disease is suspected, if the abdomen becomes full (*ascites*) disease of the liver is suspected, whereas dropsy of the feet and ankles results usually from heart disease. In all cases of dropsy there may be albumen in the urine. There is not often much pain in heart disease unless the pericardium, *i.e.*, the sac round the heart is involved; in such conditions, named *pericarditis*, there may be a collection of fluid pressing upon and interfering with the action of the heart, as occurs in rheumatic fever, or a collection of pus as occurs in pyæmia. The dyspnœa or difficulty of breathing may be very marked, especially in pericarditis or valvular disease accompanied by congestion or inflammation of the lungs, the slightest effort bringing it on. The difficulty of breathing is greatly increased by exertion and may come on in paroxysms. The patient is often more comfortable when propped up in bed or sitting up in a chair. Disease of the aortic valves is much more common in men than in women and is the most fatal form; disease of the mitral valves is not generally fatal and death rarely occurs from it, being due, on the other hand, to congestion of the lungs or to dropsy. The mental symptoms are also more marked in aortic disease, the patients being more irritable, restless and sleepless. Disease may attack the veins, causing blood to clot in them, and when the valves in the large veins of the legs give way varicose veins and varicose ulcers result. Disease attacking the large arteries is serious and may cause *angina pectoris* with sudden pain and spasm, as also aneurism, the whole or part of the artery dilating and giving rise to symptoms of pressure, eventually bursting and causing sudden death. The nurse's duties in mental cases with heart disease are to avoid any unnecessary strain, to keep the patient calm, to see that the meals are not too heavy and the stomach not over-taxed and over-loaded. Avoid exciting conversation or conveying bad news, or any emotional disturbance. It is often found best not to mention their heart disease to mental patients. Such patients must not be opposed or thwarted, and no discussion of their delusions or views is to take place, as it may bring on excitement with faintness or collapse. Their irritability and restlessness must be tolerated, and they should avoid all haste, over-strain, hurry, and impatience. Rest in the most comfortable posture is to be observed as much as possible, and the horizontal position on a bed or couch is the least effort for the heart and the one in which the circulation is easiest and least taxed. If the patient faints, the head should be low; pain can be relieved by warmth or a counter irritation. Dropsy needs special instructions and treatment. Heart affections are commoner among insane patients than among the ordinary population, and the nurse is expected to be familiar with the various applications of massage and bathing as already referred to in a previous lecture and as used in the Schott or Nauheim treatment for cardiac cases.

### Foreign Nurses in Japan.

BY A MEMBER OF THE ORDER OF SPANISH-AMERICAN WAR NURSES.

An idea may become prevalent that the American nurses went to Japan more for pleasure than for work. As a member of the Order of Spanish-American War Nurses, and being in direct communication with them, I should like to state facts.

The President of the Order of Spanish-American War Nurses—from which the nurses in Japan were drawn—is Dr. Anita Newcomb McGee, a fully-qualified medical practitioner, and a few extracts from the record of her work in Japan may not be uninteresting. Summing up what has been done since her arrival in March, she says:—

"After due deliberation it was decided that our party would be of greatest service at the Army Reserve Hospital of Hiroshima. This is a city of 122,000 inhabitants at the mouth of a river emptying into a bay of the Inland Sea of Japan. It is the place from which almost all the transports sail. It is also the point to which virtually all the sick and wounded soldiers are brought by the hospital ships. On the opposite side of the inland sea is Matsuyama, where the Russian prisoners are detained.

"Our hospital included several sections, called 'divisions,' located in different parts of the city for convenience of site, and as a precaution against fire. The 'main division' is the permanent hospital of the garrison in peace times, while the other divisions consist of numbers of temporary wooden wards, each of about 66-bed capacity, much like the wards of our Joshua Simpson Hospital, built near Fort Monroe in 1898. The main division has six wards of different sizes and plans, and many detached buildings for administration, operations (with surgical dressings and bath), bacteriological and chemical laboratory, nurses' dressing-rooms, kitchen, convalescents' recreation-rooms, store-rooms, etc. It is here that our party is stationed, one nurse working in the operating-room, and two in each of the three largest wards. One nurse is on each of the hospital ships belonging to the Red Cross Society, running between Manchuria and Hiroshima.

"It is the policy of the Japanese to follow the latest developments of military surgery, according to which the aseptic first-aid dressing is applied to a wound as soon as a soldier falls, either by someone belonging to the medical department or by the men themselves. These dressings are examined, and, if necessary, reapplied at the dressing station, after which the wound is not touched nor the dressing changed, unless conditions require it, until the patient reaches the base hospital, which, in the present war, is the one at Hiroshima. Only in rare cases does a wound require immediate operation, and experience has shown the extreme danger of operating under field conditions, when suppuration is so apt to occur.

"All wounded are transferred to a hospital ship as promptly as possible, and the work of the male nurses and attendants in Manchuria comprises the transportation and feeding of patients and, when possible, taking off the men's clothes and substituting the stout cotton kimones which are worn in place of our night shirts or pyjamas. Of course a broken limb is put in a splint, but the rule against touching a wound is so strict that only in critical cases is it done.

"The consequence of this policy is tersely put by Chief Surgeon Fugita, who said to me: 'Surgically speaking, Hiroshima is the front. It lacks only the smoke and noise of battle.'

"I think the above statement will make it clear why there are no women nurses in Manchuria, since the amount of physical labour required is very great, while the amount of trained nursing is almost nothing. On each of the hospital ships there are 22 male and the same number of women nurses, but the "cream of the work" for the nurse (so to speak) is at the main division of the Hiroshima hospital, where most of us are. Sick and lightly wounded men are sent to the other divisions.

"Captain Tanaka is the commanding officer of the main division, and performs all the operations, assisted by the ward doctors. I must say a word here of this surgeon's admirable work, which is praised by all who have been admitted to his operating-room. Amputations and deaths are both quite unusual.

"The nursing of this division is all done by women who were trained under the Japanese Red Cross Society, and who have been called from their homes to serve in the war. They have the quiet gentleness which is a well-known characteristic of Japanese women, but their training as nurses seems to be on a different basis from that established in our country, and their experience appears to be much less than our army nurses possess. For example, most of the nurses in this hospital are from cities or towns where they had one year of lectures and one year in a hospital; but since their graduation (which may have been many years ago) they have had no experience in nursing. The detachment of nurses to which we belong is from the Tokyo Red Cross Hospital, and had longer training, but it is not customary for Red Cross nurses to continue their work after leaving the hospital, so some of these are married, or living with their parents, or have left industrial or other schools where they were taking up other studies.

"Two or three of our nurses and six to nine Japanese nurses are placed in each of three wards of different sizes. The division of work in the wards has adjusted itself easily, and no difficulty of serious nature has arisen on account of the difference of language or for any other reason. All of our nurses have learned the most necessary words and phrases, and seldom have to call for help from the nurses who speak English.

"Miss Kemmer has shown great ability in acquiring the language, and uses it to advantage in the operating and dressing pavilion, where she assists daily. All dressings, except in cases where it would be dangerous to move the patient, are done in this pavilion, and our nurses are invited to accompany their patients there whenever their work permits.

"I recently asked permission for the party to have turns in working on hospital ships, and this was kindly granted by the Minister of War, who added that I might also go on the ships as often as I wished. Each of these ships is intended to carry 225 patients, but sometimes it is necessary to take many more. On the first seven voyages of the *Kosai Maru* 1,503 were carried, of whom three died on the trip; 647 of the total were wounded in battle, 96 wounded by accidents, and the rest were sick.

"Each ship is provided with an operating-room, *x*-ray room, steam disinfecting plant, and dispensary. These ships were of course fitted out under military supervision, and their work is strictly under military orders, just the same as the three other hospital ships belonging to the army, viz. the *Yokohama Maru*, *Rosetta Maru*, and *Rohira Maru*, which ships also carry doctors and nurses furnished by the Red Cross Society."

From a letter written by Miss King, a Philadelphia nurse of the party, I quote the following:—

"We expect to sail on the *American Maru* from Yokohama on September 20th. We did not intend to go until the last day of October, but Dr. McGee wished to go a month earlier. At first we felt that we ought to remain, as the heaviest of the work is coming, but the Red Cross Society say we have done what they intended our coming to do, namely, to teach a lesson of independence to the Japanese women, and also to show to the nation that they have the sympathy of the American people. Not being acquainted with their language, which, by the way, is the worst problem I ever tried to solve, has hindered us a great deal in doing more work; but what we could do we have done in the best way we knew, conforming as much as possible to the Japanese way of doing things. The Red Cross Society as an organisation is almost perfect. We have been amazed to see how perfectly everything has worked, and how much is done for the comfort of the sick and wounded. The women nurses are well trained, and they certainly are faithful and tireless workers. Every Japanese soldier considers his wound received in battle a decoration; and everyone else treats him as a hero, even if he is a very common, ordinary individual. We have enjoyed caring for them very much, they are so quiet and patient; no matter how ill they are, you never hear a groan or murmur.

"We were granted permission to make a trip on the hospital ship to Manchuria; Miss Kratz and I went on the *Haknai Maru*, leaving here on July 24th. We arrived in the Bay on the 28th, and after a short time we anchored at Dalny. Our patients had not arrived, so we received permission to go on shore on the 29th. It was raining and we found the Manchurian mud very sticky. We walked round and viewed the ruined houses and in the five hours we were there we saw nothing but Japanese soldiers, Chinamen, and a very few women. We were much amused when the Chinese asked if Miss Kratz and I were Russian prisoners?

"The city shows evidence of being well planned, as the streets are wide and usually paved in the residential portion. The houses are of brick and stone, and some look very imposing, having extensive gardens enclosed by high walls. Scarcely one house is in good condition as the Russians burned a great many, and the Japanese bombardment destroyed what the Russians did not.

"The Dalny Hotel and the City Hall are nothing but walls, the interiors being completely destroyed. Examining one of the trains, we found that the locomotive was English. While at Dalny we heard heavy cannonading, as Port Arthur is not more than 10 miles across the country, and we also heard that the Japanese had captured a portion of the hill lying to the north-west of Port Arthur. At the time they had been fighting for three days—from the 26th to the 28th.

"On the 30th we took our patients on board and started for home. Our return was without incident. We arrived in Hiroshima on August 3rd, making the trip in 10 days. We have nothing but words of praise for the nurses and all the people on board ship for the careful and expeditious way in which the sick were handled and afterwards cared for. We carried over 300 sick and wounded, and had only one death. This letter is written on Japanese paper, and is rather blotted I fear, but the Japanese use a brush to write with, and our pens are not adapted to the soft paper."

### Nursing at the Pasteur Hospital in Paris.

"I came over to see how nurses are trained in England," said Sister Mary Catherine, the Lady Superior of the Pasteur Hospital in Paris, when I called upon her last week, at the French Convent of the Reparation in Beaufort Street, Chelsea. Sister Mary Catherine was the only nursing sister who accompanied the French medical men to London for the late Congress; and she certainly made the very utmost of her short stay here, visiting with untiring energy many of our hospitals.

"I have," she said, "been to St. Thomas's Hospital, University College Hospital, Guy's, the Children's Hospital in Great Ormond Street, and I am just off now to St. Bartholomew's. All my time is filled up, but I hope to be able to see the Victoria Children's Hospital at Chelsea, and at least one fever hospital, before I leave."

"May I ask you to give me your impressions of what you have already seen?"

"I am very pleased with everything I have seen. One thing that has struck me particularly is the extreme comfort, almost luxury, of your hospitals. I think that both patients and nurses seem very well cared for, and the nurses, on the whole, look very healthy and happy. I have had several interesting chats with matrons, and also with some of your eminent medical men. I am very anxious to see and to learn all that I can."

"Is it your intention to adopt any particular nursing system you may have seen and liked here?"

"That is a difficult question to answer, because of our religious order. But I hope in future to be able to train some of our young sisters more as your probationers are trained. I am very hopeful about this, because I think that sisters of charity can be taught to nurse quite as well as other women."

"Will you kindly tell me a little about your hospital and the duties of the nursing sisters?"

"Our hospital, which is in the Rue de Vaugirard, was opened four years ago for infectious diseases, to the memory of Louis Pasteur. There are 60 beds, and we have two resident medical men. We sisters are from the community of St. Joseph de Cluny. There are at the hospital 28 of our order, 12 of whom are nursing sisters. The duties of the nursing sisters are, naturally, to wait upon the patients and carry out the doctors' instructions. We have no wards; each patient has a cubicle. We have a great many diphtheria cases, and sometimes, of course, hydrophobia. Our sisters—three of whom are Irish—have all been trained in the hospital, and, as most of them have been there since it was opened, they are very experienced by now; and they have lectures from the medical men."

Then, as I glanced at the Superior's dress and veil, I thought of uniforms, and asked how they managed to retain their religious garb in an infectious hospital.

"Indoors," she answered, "we wear white washing clothes entirely; even our veils are white."

"Then with regard to the hours of duty?" I inquired.

"The day nursing sisters are on duty from 5.30 A.M. till 9 P.M. The recreation hours daily are from 12.30 to 1 30, and from 7 to 8.30 P.M.—but these are not certain. Our night sisters are on duty from 9 P.M. to 5 A M. only. We have two or three night sisters, according to the number and severity of the cases, one acting as night superintendent. The sisters are on night duty one night a week only. All of our nursing sisters have leisure for their devotions, and a certain amount of time for study. But we work very hard," she said, "harder than your nurses in England, and we have no holidays."

"Do the nursing sisters live in the hospital or in a home?"

"They live in a separate building. I much admire many of your hospital nurses' homes. But we have all we require," the Superior added, gently.

Then she looked at the watch she had been holding in her hand all the time, and said, "I am afraid I must be going now; my last minute is up."

### The Development of Workhouse Nursing.

BY MAJOR R. F. BALLANTINE.

In looking back during the past quarter of a century over the many and varied changes which have taken place in society generally—more especially those referring to the condition and welfare of the masses—one is struck by the wonderful progress of the nursing profession. This is more apparent to those brought into contact with public institutions, and the treatment of people in them, and whether it be in infirmaries, lunatic asylums, or in the many workhouse and other hospitals spread broadcast throughout the land, the care and attention to poor suffering humanity is now found to be enlisting the sympathy and kindly consideration of those who are officially connected with the administration of these various centres of usefulness, to a very much greater extent than formerly. In connection with no public department has there been greater progress made in this direction than in the Poor Law service—in fact, one might almost term the advance of nursing and consideration for the sick poor in our workhouses during the past quarter of a century as "startling." For this, one must admit, the amazing change in the feeling of the more fortunate members of society towards those who, less fortunate than themselves, have been compelled through sickness and physical and mental disability to seek the help of the Poor Law, has a great deal to do.

### Instances of Altered Conditions.

From personal observation I can give wonderful instances of the altered nursing conditions of certain institutions as compared with their state 28 years ago. In one Poor Law institution in England in 1878, prior to the introduction of properly trained nurses, the nurses and attendants then employed in attendance on the sick patients (numbering say 600), were practically untrained, and largely consisted of inmates of the workhouse. The same infirmary to-day, removed to a more salubrious position outside the large manufacturing town in which it was situated, has a thoroughly up-to-date, highly-trained, and efficient staff of 100 nurses, and is one of the best training schools in England, sending out year by year nurses to every part of the world. Another similar instance, known personally to me, is a workhouse infirmary in England, which in 1878 had not a single trained nurse to look after its 250 sick patients, and has now 30 fully-trained and competent nurses so well fitted and equipped for their important duties that certificates of efficiency are granted them on the termination of three years' training on the authority of the Local Government Board. It may be taken for granted that many similar instances like these could be given, all showing the rapid advance of nursing in our Poor Law institutions. At times I have been much amused at the remarks of some of the young medical officers as to certain duties performed by the nurses in the wards under their care; these young gentlemen finding frequently on taking up their duties that this work, they, as medical men had to do was being done quite as efficiently by the nurses, as a part of their regular duties. This speaks well for the practical training they get in the proper performance of their trying work.

### Recognition of Responsibilities.

There is no doubt that the governing bodies of these institutions are year by year becoming more alive to their responsibilities in connection with the poor people they have under their care, and in providing suitable accommodation for them, freely avail themselves of the advantages and facilities granted them by Government in getting the necessary funds for the purchase of sites and for buildings, but one is compelled, nevertheless, to admit that to the Local Government Board and their policy, as expressed by their inspectors throughout the country, the great success and improvement in the nursing profession is primarily due.

### The Workhouse Infirmary Nurse.

Then, again, to the observer who can remember the training of nurses in the past and compare that training with the trying examinations, lectures, demonstrations, etc., to be undergone by the probationer of the present day before she is able to get her certificate of efficiency, the difference is most marked, and shows in a conclusive way the advantages gained by the general public in being able to utilise such valuable agencies for good. We cannot but admire the private nurse as we see her brought in as an assistant to the medical man in almost every case of a serious character in everyday life; or the district nurse, flitting about in our densely populated slums in our smoky and begrimed towns, or in the villages in the country, on their peaceful and health-giving mission, bringing comfort and happiness into the homes they visit; or the nurses in our large general hospitals, ever ready to carry out the instructions and directions of the doctor. But I have been more especially concerned with the workhouse infirmary nurse, whose life is passed during her term of service in daily contact with the seamy side of human nature, in alleviating the pain and suffering of these poor people committed to her care, and I desire especially to testify to their value. It is also a great advantage that in these institutions there are now a large number of fully qualified and efficient nurses specially trained as midwives. Every year hundreds of these obtain the certificate of the London Obstetric Society, enabling them to follow this branch of their profession specially authorised and equipped.

### Crumpsall Nurses.

It is pleasing to note the *esprit de corps* which exists in connection with the special infirmary or training-school one has been connected with. For instance, the other day I went to see a picture now being exhibited in the North of England, 150 miles from Manchester, and found a couple of Crumpsall trained nurses looking on, one working in that locality, the other *en route* to the South of England, to fill an important position there. We had a talk together, and agreed that there could not possibly be a better training-school than Crumpsall! I opened my letters a day or two later, and found a letter from a friend informing me how pleased and thankful she is that her son, who is suffering from a serious illness in Freetown, Sierra Leone, West Coast of Africa, is being nursed by a Crumpsall-trained nurse, as my correspondent pathetically puts it, "trained by my late dear sister." The reference is to my highly esteemed friend the late Miss Hanan, who did so much for nursing in the past. In connection with my voluntary temperance work, I travel from time to time to different parts of the Kingdom, and frequently find some of the old Crumpsall nurses waiting to have a chat with me at the end of the meeting. Although they are comfortable and happy in their work, they always revert in their conversation to the happy days at Crumpsall! And what is true in respect to Crumpsall is no doubt equally true concerning Liverpool, Birmingham, Chorlton, or any of the other large and important training-schools in the metropolis in connection with the nursing of the sick poor. As an old Army man this aspect of the training-schools appeals to me in a very marked degree.

The Properly-Trained Workhouse Nurse.

I should also like to give my personal experience respecting the qualifications of the workhouse infirmary-trained nurse, and those nurses who have been trained in general hospitals, outside the Poor Law, some of whom have been imported to fill up vacancies in the staff. I am bound to assert that for a thorough all-round training, the properly trained workhouse nurse has the advantage. Formerly major operations in surgery were few in workhouse infirmaries, and frequently outside professional help had to be called in. But nowadays they are regularly done by the staff of the infirmary, and the scene in the operating-theatre of one of these training-schools, fitted with the most up-to-date appliances, sterilisers, and other antiseptic methods, the operating surgeon clad from head to foot in specially prepared garments, with the crowd of nurses, eager and intelligent, looking on and noting the course of the operation, is one never to be forgotten. I know it has been frequently stated that it was the want of experience in surgical cases which placed the Poor Law nurse in a secondary position to the nurse in a general hospital, where, it was contended, greater experience was obtained. That disadvantage has been removed, and I cite the fact that the Poor Law nurse is called upon to attend upon patients suffering from greater variety of ailments to justify my conviction that for a "general" training—that is, the training for general need—the Poor Law nurse has the advantage. Moreover, in many of the general hospitals a large proportion of the minor acts in connection with ward work are done by students preparing for the medical profession, which prevents the nurses from obtaining that proficiency which practice alone can give. I conclude with another satisfactory point in connection with the Poor Law nurse, namely, the decided increase in the remuneration paid to her. For many years £20 a year was considered a good salary, but now in many infirmaries salaries of £32 to £34 are cheerfully given.

### Appointments.

British Seamen's Hospital, Constantinople.—Miss Florence Alice Sparrow has been appointed nurse. She was trained at Bethnal Green New Infirmary, and has since been assistant surgical nurse at the Great Western Railway Hospital, Swindon, Wilts.

Grove Hospital, Tooting.—Miss F. A. Webber has been appointed charge-nurse. She was trained at Hackney Infirmary, and has since been charge-nurse at Brook Hospital, Shooter's Hill, and ward sister at St. Leonard's Infirmary, Shoreditch.

Kingston-on-Hill Union Infirmary.—Miss C. M. Simkins, Miss J. Wilson, Miss E. M. Barber, and Miss G. Barter have been appointed additional ward sisters. They were trained at Kingston Union Infirmary. They all hold the L O S. certificate.

Maidenhead Union Infirmary.—Miss Jane Cook has been appointed assistant nurse. She was trained for two years at St. Peter's Home, Woking, by the Meath Workhouse Nursing Association.

Margate Cottage Hospital.—Miss Annie Hyde has been appointed staff nurse. She was trained at Southwark Infirmary, East Dulwich, and has since been charge-nurse at the Holborn Union Schools, Mitcham, Surrey.

Mount Vernon Hospital, Hampstead.—Miss G. J. Jones has been appointed staff nurse. She was trained at St. Cross Hospital, Rugby, and has since been staff nurse at the General Hospital, Stratford-on-Avon.

Nover's Hill Hospital, Bristol.—Miss S. A. Mountford and Miss L. Hiscock have been appointed charge nurses. Miss Mountford was trained at St. Marylebone Infirmary and the Northern Fever Hospital, London, and has since been charge nurse at the Isolation Hospital, Stoke-on-Trent, and holiday sister at St. Marylebone Infirmary. Miss Hiscock was trained at Monsall Fever Hospital, Manchester, and Poplar and Stepney Sick Asylum, London, and has since been charge nurse at Leigh Joint Hospital, Astley, and private nurse at Southsea.

Savernake Cottage Hospital.—Miss F. K. Houghton has been appointed matron. She was trained at St. George's Hospital, London, where she has since been sister.

St. George's Infirmary, Fulham Road, London.—Miss J. M. Avery and Miss A. E. Cawood have been appointed sisters. Miss Avery was trained at St. Marylebone Infirmary London. Miss Cawood was trained at St. Marylebone Infirmary, and has since been charge nurse in the same institution, and sister at Nottingham Infirmary.

Thetford Union Infirmary, Norfolk.—Miss Gertrude Hall has been appointed head nurse. She was trained at the Chorlton Union Hospital, West Didsbury, Manchester, and has since done private nursing at St. Anne's-on-Sea.

Ulster Hospital, Mount Pottinger.—Miss J. Hill has been appointed sister. She was trained at Victoria Hospital, Blackpool, and has since done private nursing at Northampton.

Wolverhampton and Staffordshire General Hospital.—Miss Lilian Barrow has been appointed sister. She was trained at the Liverpool Royal Southern Hospital, where she has since been staff nurse and holiday sister. She has also been sister at Torbay Hospital, Torquay, has done duty as a member of the Army Nursing Service Reserve, and taken temporary matron's duty at the Borough Hospital, Ashton-under-Lyne and the West Cornwall Miners' Women's and Children's Hospital, Redruth. She holds the L.O.S. certificate.

Workhouse Infirmary, Milton, Portsmouth.—Miss Maude Rogers has been appointed assistant matron. She was trained at Birmingham Infirmary, and has since been nurse at the British Hospital, Algiers, and sister at Poplar and Stepney Sick Asylum, London.

### "The Life of Florence Nightingale."

Very appropriately on the 50th anniversary of the departure of Florence Nightingale for the Crimea, Messrs. S. H. Bousfield and Co. published her Life by Mrs. Sarah A. Tooley, author of "The Personal Life of Queen Victoria." Mrs. Tooley, who dedicates her work to Lady Herbert of Lea, has been fortunate enough to secure 22 excellent illustrations, including several portraits of Miss Nightingale. Starting with the birth of the baby, who was called "Florence," because she was born in the Italian City, she proceeds to the days at Lea Hill, Lea Hurst, and Embley, where the little girl showed her early characteristics by tending with loving care her dolls, who were always either sick or convalescent, but never well, and by saving from hanging her first patient, a shepherd's dog. The poor beast's leg had been so injured that the shepherd was preparing to kill him when Florence, with the advice and help of the clergyman, took the mangled member under her care, and to the joy of its owner restored it to working powers again.

The nursing instincts of Miss Nightingale were subsequently quickened by her meeting with Elizabeth Fry, whose account of her travels abroad, and of her small training home for nurses in London, greatly impressed her. Before her departure to Kaiserswerth, on which we need not dwell, because of the full description of that institution given in our issue last week, she had spent some months in the leading hospitals of London, Edinburgh, and Dublin, and of France, Germany, and Italy. Upon her return from Kaiserswerth she published a little history of the hospital, and being asked for a copy of one in September 1897, Miss Nightingale wrote in pencil the following letter :—

"Messrs. Dubau,—A gentleman called here yesterday from you, asking for a copy of my *Kaiserswerth* for, I believe, the British Museum. Since yesterday, a search has been instituted—but only two copies have been found, and one of those is torn and dirty. I send you the least bad-looking. You will see the date is 1851, and after the copies then printed were given away, I don't think I have ever thought of it. I was twice in training there myself. Of course, since then hospital and district nursing have made great strides. Indeed, district nursing has been invented. But never have I met with a higher love, a purer devotion than there. There was no neglect. It was the more remarkable because many of the deaconesses had been only peasants—(none were gentlewomen when I was there). The food was poor—no coffee but bean coffee—no luxury but cleanliness.

"Florence Nightingale."

After a period of waiting, during which Miss Nightingale was twice seriously ill—once in Paris and again after she had organised the Harley Street Home for Sick Governesses—the war between England and Russia broke out, and Sir William Howard Russell appealed in the *Times* for some women to minister to the sick and suffering in the East. Here Mrs. Tooley recalls the fact that Sidney Herbert, then Secretary of State for War, determined to respond to it, if Miss Nightingale would consent to lead the nurses and wrote to her, "There is but one person that I know of who would be capable of organising and superintending such a scheme. Would you go out and supervise the whole thing?" On that memorable 15th of October Miss Nightingale wrote to Sidney Herbert offering her services in the hospitals at Scutari, and their letters crossed.

"It is characteristic," observes the author, "of Miss Nightingale's method and dispatch that only a week elapsed from the day on which she made her great resolve to go to the help of the wounded soldiers until she had her first contingent of nurses in marching order. She was a 'general' who had no parleying by the way, but worked straight for her ultimate object, and she possessed also the rare faculty of inspiring others to follow her lead."

As Mrs. Tooley says,

"The first appeal for nurses did not bring satisfactory applicants. Kind, generous, and sympathetic women volunteered by the score, but Miss Nightingale and her friends felt that they were dealing with a crisis of urgency. There was no time to start ambulance classes and train candidates. It was an imperative necessity that the nurses should start without delay, and therefore they must have been already trained for the work. In the emergency Miss Nightingale applied to both Protestant and Roman Catholic institutions for volunteers. This caused a good deal of adverse criticism. The 'No Popery' cry was raised, and zealous clerics inveighed against Miss Nightingale as a Puseyite who was bent on perverting the British soldier to papacy. She certainly was at the time more engaged with the bodily than with the spiritual needs of the soldiers. Nurses were required, not religious instructors."

Miss Nightingale and her 37 nurses, called by Kinglake the "Angel Band," set forth under cover of night :—

"Only a few relations and friends stood on the platform of the terminus on that October evening when Florence Nightingale bade farewell to home and kindred and started on her great mission, the magnitude and difficulty of which she had yet to discover. Quietly dressed in black, plain as a Quakeress, she was yet a striking figure. As the last handshake was given and the last farewells said her beautiful face retained its calm demeanour and was illumined by a sweet smile. Ever thoughtful for others, her chief wish was to spare her nearest and dearest, who had yielded a hesitating consent to her undertaking, from anxiety. None knew better than herself the perils which lay in those far-off Eastern hospitals."

In France, however they did not escape observation—

"It was a circumstance to arouse French enthusiasm, and when Miss Nightingale and her nurses stepped ashore they were met by a stalwart company of Boulogne fishwives, a merry and picturesque band in snowy caps and gay petticoats, who seized trunks and bags and almost fought for the privilege of carrying the luggage of *les sœurs* to the railway station. They would accept no pay, not a *sou*, and they bustled along with their brawny arms swinging to straps and handles, or with boxes hoisted on their broad backs, chattering of "Pierre" or of "Jacques" out at the war, and praying the *bon Dieu* that if he suffered the sisters might tend him. The tears streamed down many of the old and weather-beaten cheeks when they said adieu. They claimed but one reward, a shake of the hand, and then as the train steamed out of the station they waved their hands and cried *Vivent les sœurs!*"

On their arrival at the Scutari Hospitals they found that

"the sufferers had heard of the coming of the sisters, but the news seemed too good to be true, and when Miss Nightingale went her first round of the wards, accompanied by members of her devoted band, 'Tommy's' heart was full. One poor fellow burst into tears as he cried, 'I can't help it, I can't indeed, when I see them. Only think of English women coming out here to nurse us! It seems so homelike and comfortable.'"

The author then gives a description of the Barrack Hospital, which was a scene of filth, pestilence, misery, and disorder, and recites the experiences in the Crimea of Miss Nightingale, who found that with heaps of stores the patients were all starving :—

"There was an organising brain, however, at work in that dreadful Barrack Hospital now, and *within ten days* of her arrival, in spite of the terrible influx of patients which taxed her powers to the utmost, Miss Nightingale had fitted up an impromptu kitchen, from which 800 men were daily supplied with well-cooked food and other comforts."

During Miss Nightingale's labours at the seat of the war Queen Victoria, it is well known, followed her movements with unflagging interest, and on December 6th, 1854, her Majesty wrote to Sidney Herbert—

"Would you tell Mrs. Herbert that I beg she would let me see frequently the accounts she receives from Miss Nightingale or Mrs. Bracebridge, as *I hear no details of the wounded*, though I see so many from officers, etc., about the battlefield, and naturally the former must interest *me* more than any one. Let Mrs. Herbert also know that I wish Miss Nightingale and the ladies would tell these poor, noble wounded and sick men that *no one* takes a warmer interest or feels *more* for their sufferings or admires their courage and heroism *more* than their Queen. Day and night she thinks of her beloved troops. So does the Prince. Beg Mrs. Herbert to communicate these my words to those ladies, as I know that *our* sympathy is much valued by these noble fellows. Victoria."

The whole story of Miss Nightingale's heroism and devotion, how she lost her nurses by cholera, how she saw Sebastopol from the trenches, how she was suddenly taken with the worst form of Crimean fever, how, during her convalescence, she started a memorial at Scutari to the fallen heroes, how she established a staff of nurses in the hospitals on Balaclava Heights, how she returned to England, arriving *incognita* as "Miss Smith," is graphically told by the author.

Although in weakened health, Miss Nightingale volunteered in 1857 to go out and nurse the soldiers engaged in the Indian Mutiny, but her offer was not accepted, as there appeared to be no need. Upon that portion of the work, which deals with the establishment of the Nightingale School for Nurses, we need not touch; nor yet upon the splendid work which she did in connection with the Liverpool School and Home for Nurses. Fourteen months after the death of Lord Herbert she wrote to a correspondent the following letter:—

"October 22nd, 1861.

"Dear Sir,— . . . In answer to your kind inquiry, I have passed the last four years between four walls, only varied to other four walls once a year; and I believe there is no prospect but of my health becoming ever worse and worse till the hour of my release. But I have never ceased, during one waking hour since my return to England five years ago, labouring for the welfare of the army at home, as I did abroad, and no hour have I given to friendship or amusement during that time but all to work. To that work the death of my dear chief, Sidney Herbert, has been a fatal blow. I assure you it is always a support-giving strength to me to find a national sympathy with the army and our efforts for it—such a sympathy as you express.

"Believe me, dear sir, sincerely yours,

"Florence Nightingale."

Forty-three years have passed since this letter was written, but age has not dimmed the mental faculties of the writer, nor has illness prevented her from taking an active interest in movements for the benefit of the community, and especially of nurses. In conclusion we quote an incident which shows how Miss Nightingale, at 84, pays honour to the women of a younger generation, who are now bearing the burden and heat of the day.

"'Will you give me your blessing?' said the Superintendent of a benevolent institution to her recently, when taking her leave. 'And you must give me *your* blessing,' replied Miss Nightingale, as she took her hand. On another occasion she said to the same lady, after listening to an account of good work going successfully forward, 'Why, you have put new life into me.'"

### Jubilee "Florence Nightingale," 1854–1904.

When the thunders of war awoke the far Orient
With slaughter and bloodshed on land and on sea,
The cries of the wounded, the groans of the dying,
Were borne on the breeze to the land of the free.
The women of England were shocked at the suffering,
And quickly resolved to relieve them they'd go,
Led forth by a heroine to face every danger,
In mercy to succour alike friend and foe.

Oh, cruel the pangs by warfare so riven,
Of the brave who soon die of neglect and disease:
Their agonised prayers once ascended to Heaven
Caused angels to offer their sufferings to ease.
Then humanity's star gleamed bright in the firmament,
Raised the emblem of mercy whose light ne'er will pale:
It increased in radiance, and at once shed a halo
On that noble nurse heroine "F. Nightingale."

The world viewed with wonder the step she had taken,
All hailed the results with delighted surprise:
Her noble example compelled emulation,
And the neutral Red Cross at once did arise.
She strove to allay the poor soldiers' deep anguish,
Attended their wounds and nursed them in pain,
Cheered the hearts of the hopeless; and the maimed and the crippled,
From her took fresh courage to face life again.

There were many brave heroines in all the past ages,
Who have died for their country, religion, and laws:
They emblazon the annals of history's pages
And merit the patriot's greatest applause;
But none of them shine with so pure a reflection,
Or can claim of the nation such homage and fame,
As the woman who first trained nurses by mercy,
Shedding lustre and glory on woman's fair name.

Note.—The above tribute has been sent us by a patient, Frederick Robinson, who is confined to bed in the West Ham Union Infirmary. He writes:—Well do I remember the interest aroused throughout the country on the departure from England on October 21st, 1854, of "Florence Nightingale" and her nurses. In bringing it to your notice I hope you may not think me presumptuous in wishing topay my tribute of appreciation to the greatest benefactress of the civilised world.

### TRAVEL NOTES AND QUERIES.

By our Travel Correspondent.

Hotels in Montreux (Ida).—You do not attend to my oft-repeated requests that correspondents will tell me how much they can afford to pay instead of asking for addresses of hotels where charges are moderate. No two people probably would agree on the scale that should be called moderate. Being quite in the dark as to the amount you wish to pay, I must fall back upon my own idea of moderate charges. Six villages—Clarens, Charnex, Vernex, Glion, Colonges, and Veytaux — collectively, form Montreux. At Clarens, Hôtel Pension Ketterer, from 6 frcs.; Hôtel Pension, Verte-Rives, same terms. At Vernex, Hôtel Cygne, from 7 frcs. At Glion, Hôtel de Glion, from 6 frcs. If you remain a fortnight and more you would probably be able to arrange for lower terms. If I can help you further I shall be pleased to do so, but please be more explicit.

### A Visit to the Valencia Village Hospital, Ireland.

BY A CORRESPONDENT.

Valencia Island, county Kerry, Ireland, where I have been spending a week, is one of the sweetest spots I have ever seen. One afternoon I paid a visit to the village hospital, which is a tiny building, only making up eight or nine beds, though it treats a great number of out-door patients.

### The Matron.

The matron, a most kindly Irish lady from Cork, Miss O'Keeffe, has only one servant to assist her, and when she takes her well-earned holiday in the autumn, the establishment has to be closed until her return, as the funds will not allow of the engagement of a temporary matron. Miss O'Keeffe also, in her leisure moments (which are not many) visits the poor in their humble homes throughout the whole island and gives all the help she can in cases of illness or accident, which for many reasons may not have been taken into the hospital. To accomplish this she has a donkey and trap for wet and stormy weather and a bicycle for finer days.

### Origin of the Hospital.

The building which formed the nucleus of the present hospital was a wooden one, and was originally used by the Anglo-American Telegraph Company, when the *Great Eastern* steamship came to lay the cable between the hemispheres. When it was no longer required by the company, they were petitioned to hand it over to be used as a hospital, and they readily agreed to present it for that purpose to the late Knight of Kerry and Dr. Blennerhassit, the then doctor of the district. It was moved to the present site, a gift to the managing committee, by the late Knight of Kerry in 1871. The wooden building was in use until 1884, when after much difficulty sufficient money was collected to build the present hospital consisting of two wards—male and female—matron's bed- and sitting-room, servants' room, bath-room, etc., and kitchen. In the beginning of the 'nineties it was proposed to enlarge the building by adding a private ward and sitting-room. The late Dowager Lady FitzGerald undertook to collect sufficient for this purpose but unfortunately died before she was able to accomplish it and it was not until 1899 that the addition was completed as a memorial to her.

### Instituting an Isolation Ward.

In 1880 and 1881, when the soldiers were stationed in Cahirciveen, a little town on the mainland, they were quartered in wooden huts, and upon their departure the officers' mess hut was purchased by public subscription and placed in the hospital grounds for an isolation ward. This has since been sheeted with corrugated iron, and has proved of great service in checking the spread of infectious diseases. The Anglo-American Cable Company contributed generously to this object; indeed they have always shown themselves deeply interested in the success of the hospital, on condition that two beds should always be at their disposal in case of fever or other infectious cases occurring among their staff.

### The Friends of the Hospital.

The matron is a good friend to the young men of the neighbourhood, and has formed the "Foresters' Club," which meets in the hospital kitchen in the evenings for games and recreation, and in return the members undertake the care of the garden, the tending of the donkey, and all the other odd jobs. Every year they get up a raffle; on one occasion they persuaded some one to give them a sheep, at another time a donkey, and so on, and the few pounds that these bring in are devoted to the good of the hospital. Every one in the neighbourhood of the hospital appears to contribute in kind if not in money, though many are able to do both. The Knight of Kerry in the list of gifts presented in 1903-1904 is mentioned as providing hay for the donkey, and beef tea—a strange combination! A lady contributed a "door scraper, mattress, carpet, and vegetables"; another, "two tapioca puddings"; whilst a firm at Tralee paid the carriage on goods to Valencia for the use of the hospital. But the most strange to English eyes are the contributions of fuel, the "Donkey-rail turf," the "Mule-rail ditto," and the "Horse-rail ditto." A ton of coals from another friend is a less remarkable but equally valuable item.

### Treatment for Rheumatism.

The Tallerman treatment for the cure of rheumatism is in use at this hospital, Lady Fitzgerald defraying nearly all expenses connected with it. The late Mr. Tallerman presented the apparatus to Lady Fitzgerald on condition that it should only be used for necessitous patients. Owing to the damp climate rheumatism is, of course, very prevalent, and the treatment appears to be highly beneficial, the matron telling me of many cases in which a wonderful improvement had been effected.

I had a chat with the honorary secretary, who is the rector's daughter, and learned that their most pressing wants for the moment were an operating theatre and a small mortuary. Besides these, I found that there were many minor comforts that would be very acceptable, as the whole establishment is of necessity carried on with the strictest economy, and there is no money to spare for any of the small luxuries that one is accustomed to find in all our large London institutions.

### Everybody's Opinion.

### THE STANDARD OF PRIVATE NURSING.

"An Ordinary Nurse" writes: Will some reader of The Hospital give her opinion of private nursing as compared with that of hospital wards? I think it reaches a higher standard as regards the individuality of the nurse. We have, of course, a different class of people to deal with in private practice, and our sympathies are called forth as well as our abilities and comprehension of existing circumstances. We have also to depend in many ways on our own resources; whereas, in the wards, we have so many "cases," and the routine does not permit of our spending the time with each that we should when treating only one. Moreover, we have so many to appeal to in the matter of treatment that we are apt to become mechanical. I am not disparaging the good and noble work done in our hospitals, but simply wish to point out that private nursing should not be looked down upon, and, taken rightly, may tend to elevate the mind of the woman and nurse. My remarks are, however, open to criticism, and I should be glad of the opinions of others on the subject."

### FEEDING THE HUNGRY.

"A Sympathiser" writes: I am so glad that there is some idea of dividing the homes for old people who can no longer work from the workhouse proper, giving employment to the shiftless and idle. In view of the distress and starvation of London—likely to be very severe this winter—would it be possible for someone to be appointed to utilise what is left by the patients (not infectious cases of course)?—as I have heard of half pheasants, fish, and pudding being all thrown together and given to the pigs. In another case, where there is a shoot on the Thames, soup and all kinds of foods were thrown down, when many starving men were on the Embankment. Cannot something be done by deaconesses and others to utilise this food for the benefit of the starving? The Little Sisters of the Poor have shown that there is a way. I raise the point for the consideration of clever matrons and heads of hospitals.

### Echoes from the Outside World.

### Movements of Royalty.

The King arrived at Buckingham Palace from Sandringham on Monday evening, and on Tuesday paid a visit to Windsor in connection with the arrangements for the State reception of the King and Queen of Portugal in November. In the course of his visit he inspected the new cricket ground and pavilion adjoining the Long Walk, which are being prepared for the Royal Household and servants. On Wednesday his Majesty visited Woolwich, in order to inspect the Royal Horse and Field Artillery batteries forming the garrison. The Queen delayed her return to England from Denmark for a few days in consequence of the indisposition of her sister, the Empress Alexander.

### Accident to the Duke of Connaught.

On Thursday last week an alarming accident happened to the Duke of Connaught. As Inspector-General of the Forces his Royal Highness had been engaged on a tour of inspection among various Scottish garrisons, and his duties having concluded with the review of the troops in Edinburgh Castle, he set out in a motor-car for Gosford House, East Lothian, some 15 miles off, to join his wife and daughters. At Pierhill the motor-car ran into a cart, and the Duke was thrown out. The collision was very violent, and the Duke's rug was torn in pieces. His Royal Highness, who got up without assistance, was driven in another motor-car back to Edinburgh, to the North British Hotel. Upon examination by the surgeons in attendance, it was found that he was suffering from a scalp wound and an injury to the left ear. Trained nurses were summoned, and at 10.15 the Duchess of Connaught arrived from Gosford by motor. A great crowd assembled in the vicinity of the hotel and eagerly awaited the issue of the last bulletin, which announced that the Duke was proceeding excellently and feeling much better. His Royal Highness continues to make steady progress towards recovery. Telegraphic inquiries respecting his condition have been received from all parts of the world. The Duchess of Connaught has remained in close attendance upon her husband since the night of the accident.

### The Throne of Saxony.

King George of Saxony died early on Saturday morning at his summer residence, Pillnitz Castle. His Majesty was nearly 74 years of age, and only succeeded his brother, the late King Albert, in June, 1902. He was a distinguished soldier and one of the few surviving field-marshals who took part in the campaign of 1870, when he commanded a division of the Army of the Meuse under his brother, who was then Crown Prince of Saxony. He subsequently became commander of the 12th Saxon Army Corps, in which capacity he displayed great abilities as an administrator. He is succeeded by his eldest son. By command of King Edward the English Court will wear mourning for three weeks for the late King.

### The Battle of the Sha-ho.

During the last ten days fighting on a terrific scale has been taking place in Manchuria. Marshal Oyama has furnished to the Japanese Government a detailed account of the operations of all the three Japanese armies on Tuesday, Wednesday, and Thursday, which are generally to the effect that, after hard fighting, the Russians were driven back all along the line, and that movements for enveloping their wings were in successful progress. Captures of over 30 guns and of many prisoners are recorded, and it is affirmed that the northward retreat of several of the Russian columns has been marked by great disorder.—In subsequent despatches Marshal Oyama states that the right army has carried the key to the enemy's positions, and that throughout the entire front of all the Japanese armies the Russians were driven back to the right bank of the Hun-ho. The total Russian losses he estimates at upwards of 30,000, the number of Russian corpses already buried being 9,000. These do not include the results of the fierce engagements with the Japanese Left Army on Friday and Saturday.—General Kuropatkin, in a telegram to the Czar on Saturday, admits that on the whole his army has had to retire. He says that the troops have been fighting for four days, and many regiments have not slept for three nights. Nevertheless he has full hope in their capacity to continue the struggle.—Despatches from General Sakharoff, received in St. Petersburg, assert that on Sunday the Russians, after fierce fighting, recovered a position which the Japanese had captured and pursued the Japanese for a considerable distance, capturing 11 field guns and a quick-firing gun—Marshal Oyama has named the great action of the past week, the battle of the Sha-ho; Japanese estimates of the strength of the Russian forces engaged put them at about 200,000 infantry, 20,000 cavalry, and 950 guns.—Many incidents from the battlefield are related in St. Petersburg. While the battle of the Sha-ho was at its height, a wounded Russian officer and a handful of wounded men reported themselves to the General in Command. The General stormed at them:—"How dare you leave your men at such a moment? Back with you at once. Where is your regiment?" "Here, sir," replied the officer. "What, is that all?" the General exclaimed with horror. "Yes, sir, that is all."

### Removal of Lady Curzon.

On Sunday afternoon Lady Curzon was moved from Walmer Castle to Walmer Place, a house in the vicinity of the Castle. The move, at Lady Curzon's desire, was decided upon 10 days previously, but was postponed owing to the occurrence of phlebitis. It was happily effected without any drawback, and the bulletin on Monday evening was to the effect that the patient had passed a quiet day, and continued to make progress. On Wednesday it was stated that since her removal to Walmer Place the improvement in her condition has been remarkable.

### Art Exhibitions.

The Institute of Oil Painters in Piccadilly, which opened on Monday, has a full and varied show. Several of the Scottish painters are well to the fore: Mr. Lavery with a "Lady in Black"; Mr. Hornel with "Blossoms," three children in grey frocks with heads close together; and Mr. Henry in a striking portrait called simply "The Brown Dress." Two French artists should not be overlooked, M. Garrido and M. Jacques Blanche. The latter's "Portrait of a Lady," in a sailor hat, with a striped white and pink dress, is especially noteworthy. "Mr. Percy Grainger," by Mr. Rupert Bunny, might have been painted by a Frenchman too. Amongst the landscapes are "The Mouth of the Exe," a capital work by Mr. Hughes-Stanton; "A Parisian Courtyard," a masterly treatment of sunlight by Mr. D. Y. Cameron; and Mr. Charles Shannon's beautiful "Romantic Landscape." At the Royal Society of British Artists in Suffolk Street, which was also opened on Monday, Mr. Tom Robertson's "Venice" should be studied carefully; and "To Pastures New," by Mr. Cayley Robinson, shows deep thought and fine painting. As well as several small landscapes by Mr. Sheard is a large figure picture, "The Village Jumble Sale." "Whispering Twilight," by Mr. Spenlove, is a delightful picture; and so in a totally different way is "The Home-coming of the Herring Fleet," by Mr. A. S. Edwards.

### A Book and its Story.

MISS MARIE CORELLI'S NEW NOVEL.*

Admirers of Miss Corelli will welcome her new book which, in spite of being over-weighted with the sayings and doings of the rural members of a population who served under the heroine, Maryllia Vancourt, the lady of the Manor of St. Rest, or formed part of the household of the rector, the Reverend John Walden, has in it sufficient elements to form a dramatic background to the sub-title of "a simple love-story." An author's note which prefaces it begs for mercy at the hands of the gentle reviewer. In consideration of this we will suspend criticism and proceed with an outline of the story which will, we hope, excite sufficient interest to send our readers to the book itself for the formation of their own verdict on its contents.

Cornwall, the county which at present forms the scene of more than one popular novel, is chosen for the situation of the parish of St. Rest, St. Est, or Saint's Rest, as it was termed, according to the source, whether local or traditional, from which its name was derived, in which the hero and heroine live. As the dedication of "God's Good man" is to the living originals of John Walden and Maryllia Vancourt, we present their counterparts, as given by the author, for possible recognition by some of our readers. "The Rev. John Walden was one of those really gifted individuals who can assume an attitude which is foreign to temperament. He was of a cheerful, even sanguine, disposition, and his countenance faithfully reflected the ordinary bent of his humour. Seeing him from a distance, the casual observer would at once have judged him to be either an athlete or an ascetic . . . A broad forehead, deeply-set, dark blue eyes, a straight and very prominent nose, a strong jaw, and an obstinate chin, a firmly-moulded mouth, round which many a sweet and tender thought had drawn kindly little lines of gentle smiling. . . . Such briefly was the appearance of one who, though only a country clergyman, of whom the great world knew nothing, was the living representative of more powerful authority to his little cure of souls than either the bishop of the diocese or the King in all his majesty. . . . As supreme dictator of his tiny kingdom and limited people, he was the sole owner of one of the smallest 'livings' in England. But St. Est, or St. Rest, was in itself so insignificant as a benefice that its present rector, vicar, priest, and patron had bought it for himself through the good offices of a friend, in the days when such purchases were possible." From the portrait of the rector we turn to his environment. The rectory, we are told, was an old fifteenth-century, lop-sided, half-timbered house, surrounded by five acres of orchard, planted with the choicest apples, cherries, plums, and pears, bounded on the south side by a high wall, where the finest nectarines and peaches in the country sunned themselves to perfection. The rector was by temperament a philosopher. He was never lonely in his retired corner of the country, for his interests and pursuits were too varied to allow of loneliness. "He loved antiquarian research, and all such scientific problems as involve abstruse study and complex calculation, but equally he loved the simplest flower and the most ordinary village tale of sorrow or mirth recounted to him by any one of his unlessoned parishioners. . . . To such a mind and temperament as his the influences of nature, the sublime lands of the universe, and the environment of existence, must needs move in circles of harmonious unity making loveliness out of commonness and poetry out of prose." The description of John Walden is concluded by the following paragraph:—"For the rest, he was physically sound and morally healthy, and lived, as it were, on the straight line from earth to heaven, beginning each day as if it were his first life opportunity, and ending it soberly with prayer, as though it were his last." This was his present condition and environment. Previously he made a name in London as an intellectual and incisive preacher, and his career at the University had marked him as a brilliant and promising scholar. He had taken the living of St. Rest partly because he knew the neighbourhood to be a veritable mine of antiquarian research and partly because since the loss of his only sister, to whom he had devoted his life up to this point, "the world became empty for him, and he felt henceforth he would be utterly companionless. For what he had seen in modern women, modern marriage and the modern ways of life did not tempt him to rashly seek refuge for his heart's solitude in matrimony." The arrival of a young and charming woman into his parish, as lady of the manor, becomes the source of some disquiet to the bachelor rector. Maryllia Vancourt, after living with a wealthy American aunt, and having spent her girlhood in wandering with her from one fashionable resort to another, had at last grown impatient of an existence that never had any attraction for her. She had been admired very much and had had many opportunities of marrying, but out of the many suitors who came forward, she had not met one in whom she had any real confidence. Apart from her own attractions, she realised that as her aunt's heiress, the attentions of her lovers were possibly as much governed by the knowledge of this fact, as by devotion to herself. One suitor's persistent and unwelcome attentions, encouraged by Maryllia's aunt, became so unsupportable that she determined to put an end to a life of servitude, by going to live on her late father's estate at St. Rest. There, in the comparative retirement, she hoped to regain some of the peace of mind to which she had been so long a stranger. Her character is a brave and dauntless one, and the first meeting between the rector and herself takes place on a corner of the estate where the agent, who for years past had had entire control of its affairs, had summoned some men who, by his direction, were to fell some fine beeches known as the "five sisters." The villagers had protested against the felling and sale of the timber, without the knowledge or permission of his mistress. Maryllia had been told of it, and the rector had been summoned to prevent the wanton destruction. While a scene of confusion is proceeding, which the rector has done his utmost to end, the arrival of Maryllia puts a new aspect on affairs. "Even while he spoke, the quick gallop of hoofs echoed thuddingly on the velvety turf, and the group of disputants hastily scattered to the right and left, as a magnificent mare, wild eyed and glossy-coated, dashed into their centre and came to a swift halt, drawn up in an instant by the touch of her rider on the rein. All eyes were turned to . . . . a figure wholly unconventional, clad in a riding skirt and jacket of deep, soft, violet hue, and wearing no hat to shield the bright hair from the fresh wind that waved its fair ripples to and fro caressingly, and tossed a spinning curl loose from the carelessly twisted braid." Maryllia had arrived in time to prevent the catastrophe. She forbids the agent to touch the trees, dismisses him on the spot and asserts her authority with an imperious dignity which impresses her rural audience greatly. The rector's after reflections on Maryllia are as follows: "Fascinating? possibly she might be—to some men. She certainly has a sweet voice, and very charming manner. . . . But there is nothing remarkable about her—she is just a woman—with a bright smile, and a touch of American vivacity running through her English insularity. Just a woman, with a *way*. Here at the beginning of "a simple love story," we must leave the two In the book itself our readers can follow its development, which meets with the usual obstacles. It comes to a tragic but happy conclusion at last.

* "God's Good Man." By Marie Corelli. Methuen. 6s.

### Notes and Queries.

**FOR REGULATIONS SEE PAGE 12.**

*Rest Cure.*

(20) Will you kindly inform me what the Rest Cure consists of, and how long a period does it take?—*Nurse.*

The details of a "rest cure" will vary according to the needs of each individual patient and the custom of his medical attendant, and the duration of treatment can only be estimated by the rapidity of improvement in each case.

*Electricity and Electric Baths.*

(21) Will you kindly inform me if I can get an inexpensive book on electricity and electric baths which also treats of *x*-rays and high-frequency current?—*Beta.*

"Natural Physical Remedies," by Dr. Hulbert, will give you the information you require. The price is 2s. 6d., and the book can be procured from the Scientific Press, Ltd.

*Hospital Training.*

(22) Can you advise me as to a suitable hospital to train as an army nurse? I am 26 years of age.

Candidates are accepted from all the best hospitals in London and the provinces. See "The Nursing Profession: How and Where to Train," for list and particulars.

Can you give me the names of a few of the best infirmaries in town where good training is given?—*N. W.*

See Burdett's "Hospitals and Charities" for complete list.

*Maternity Training.*

(23) Will you kindly tell me if it is possible to obtain a post as country district midwife without having general training?—*A. B.*

Write to the secretary, the Association for Promoting the Training and Supply of Midwives, Dacre House, New Tothill Street, Westminster, S.W.

*Weir-Mitchell.*

(24) I am greatly in need of a home for either Weir-Mitchell or similar treatment. I am not an invalid, but have come to the conclusion that a few weeks' rest in bed on milk diet would do me permanent good. Can you give me the name of any institution which would receive me, I could afford to pay about 10s. a week?—*A. T.*

We cannot recommend private homes. You had better advertise.

*St. John Ambulance.*

(25) Will you kindly tell me how I can attend the St. John Ambulance lectures, and obtain the certificate? I do not know how to set about it as I live in the country.—*Surrey.*

Write to the secretary, the St. John Ambulance Association, St. John's Gate, Clerkenwell, E.C.

*Abroad.*

(26) I should be much obliged if you could give me the address of a private nursing institution in Cairo; I cannot find one in the "Hospitals and Charities."—*Nurse F. S. A.*

We do not recommend private institutions. Write to either the English chaplain or the Consul at Cairo.

(1) Can you give me the addresses of nursing institutions at Cairo and Port Said? (2) What English hospitals are there in the Holy Land?—*I. H. D.*

(1) See reply to *Nurse S. F. A.* (2) There are English hospitals at Damascus, Gaza, Haifa, Jaffa, and Jerusalem.

*Specialist.*

(27) Will you kindly give me the name of a London specialist for diseases of the bladder, and also say if there is a hospital for the same?—*A. F. B.*

We do not give private addresses. This class of disease is treated at every large hospital.

### Handbooks for Nurses.

"A Handbook for Nurses." By Dr. J. K. Watson. 5s. net; 5s. 4d. post free.

"A Complete Handbook of Midwifery." By Dr. J. K. Watson. 6s. net; 6s. 4d. post free.

"The Nurses' Pronouncing Dictionary." 2s. post free.

"Elementary Anatomy and Surgery for Nurses." Price 2s. 6d.

How to Become a Nurse: "The Nursing Profession." 2s. 4d. post free.

The above may be obtained of all booksellers or of The Scientific Press, Limited, 28 & 29 Southampton Street, Strand, London, W.C.

### For Reading to the Sick.

REST.

When round the earth the Father's hands
Have gently drawn the dark,
Sent off the sun to fresher lands
And curtained in the lark;
'Tis sweet, all tired with glowing day,
To fade with fading light;
To lie once more the old weary way,
Upfolded in the night.

Who dwelleth in that secret place
Where tumult enters not;
Is never cold with terror base,
Never with anger hot.
For if an evil host should dare
His very heart invest,
God is his deeper heart, and there
He enters into rest.

*George MacDonald.*

If we hope for that we see not, then do we with patience wait for it.—*Rom.* viii. 25.

Can we not feel that it is well for us to pause and think of the blessedness of waiting; for it is still through long watching that at last the opportunity is found for mastering the truths towards which our hopes have been turned. It is still not unfrequently through sorrow that we gain little by little the power of insight by which the meaning of familiar facts is disclosed. It is still by silent ponderings in the solitude of the inner chamber, as in the solitude of the crowd, that we learn the lesson of communing with God. And our anxiety for results which we can measure, our restlessness under conditions which we hold to be unfavourable to our progress, our passion for excitement, tend to deprive us of these highest fruits of life. We cannot remove the conditions under which our work is to be done, but we can transform them. They are the elements out of which we must build the temples wherein we serve.

In one sense God gives nothing, while in another sense He gives all things. He requires us, that is, to make His gifts our own by using the power which He inspires. Not all at once, and not as we should have expected, and not without many delays, does that which indeed is ours become ours.

So it is that waiting itself becomes a work, and of all the promises of Scripture, none, I think, speaks with fuller encouragement to such as seem to find no fruit of labour or no scope for it, if only they wait for the Lord Who will not leave them desolate, than this: "In your patience ye shall win your souls."—*Bishop Westcott.*

To accept all the suffering that comes upon us, as sent from God, is the great secret of patience. The patience of Jesus should teach us the value of gentleness and meekness. We are so slow to believe in the power of gentleness, and yet it is the way God has always dealt with us. "I will praise the Lord because He has dealt so lovingly with me." "Thy gentleness hath made me great." We fail so often and give way to impatience because we forget God's method; we forget how gently He has borne with us, we forget that gentleness wins the day where impatience would have failed. Let us learn from the Passion the power of gentleness.

*Canon Randolph.*

